# Development of molecular assays to detect target-site mechanisms associated with insecticide resistance in malaria vectors from Latin America

**DOI:** 10.1186/s12936-019-2834-7

**Published:** 2019-06-20

**Authors:** Juan C. Lol, David Castañeda, Lucy Mackenzie-Impoinvil, Carla G. Romero, Audrey Lenhart, Norma R. Padilla

**Affiliations:** 1 Grupo de Biología y Control de Vectores, Centro de Estudios en Salud, Universidad del Valle de Guatemala (CES-UVG), 18 Avenida 11-95, Zona 15, Vista Hermosa 3, Guatemala, Guatemala; 2 Division of Parasitic Disease and Malaria, Entomology Branch, Centers for Disease Control and Prevention (CDC), Center for Global Health, 1600 Clifton Road, Atlanta, GA 30329 USA; 3 Programa Nacional de Dengue, Chikingunya, y Zika, Ministerio de Salud, Esquina Cañada Strongest, Plaza del Estudiante, Zona Central, La Paz, Bolivia

**Keywords:** *Anopheles albimanus*, *Anopheles darlingi*, *Anopheles pseudopunctipennis*, *Anopheles vestitipennis*, Insecticide resistance, Voltage-gated sodium channel gene, Acetylcholinesterase-1 gene

## Abstract

**Background:**

Malaria remains an important public health problem in Latin America, and the development of insecticide resistance in malaria vectors poses a major threat to malaria elimination efforts. Monitoring of insecticide susceptibility and the determination of the mechanisms involved in insecticide resistance are needed to effectively guide the deployment of appropriate vector control measures. Here, molecular assays have been developed to screen for mutations associated with insecticide resistance on the voltage-gated sodium channel (*VGSC*) and acetylcholinesterase-1 (*Ace*-*1*) genes in four malaria vectors from Latin America.

**Methods:**

Degenerate primers were designed to amplify a partial fragment on the *VGSC* and *Ace*-*1* genes. Wild-caught individuals for *Anopheles albimanus* (also historical samples and individuals from a laboratory strain), *Anopheles darlingi*, *Anopheles vestitipennis* and *Anopheles pseudopunctipennis* were used to optimize the PCR assays. All samples were sequenced to validate the PCR results and DNA alignments were constructed for each gene using the unique haplotypes observed.

**Results:**

Primers designed successfully amplified the *VGSC* gene in *An. albimanus*, *An. darlingi, An. vestitipennis* and *An. pseudopunctipennis*, and the *Ace*-*1* gene in both *An. albimanus* and *An. darlingi*. DNA sequencing revealed that compared with *Anopheles gambiae*, there were a total of 29, 28, 21 and 24 single nucleotide polymorphisms (SNPs) on the *VGSC* gene for *An. albimanus* (308 bp), *An. darlingi* (311 bp), *An. pseudopunctipennis* (263 bp) and *An. vestitipennis* (254 bp), respectively. On the 459 bp fragment of the *Ace*-*1* gene, a total of 70 SNPs were detected in *An. darlingi* and 59 SNPs were detected in *An. albimanus* compared with *An. gambiae*. The SNPs detected on the *VGSC* gene were all synonymous. On the *Ace*-*1* gene, non-synonymous substitutions were identified on three different codons. All species showed the homozygous wild-type *kdr* allele (coding for leucine) at codon 995 (formerly reported as codon 1014) on the *VGSC* gene, but one sample was heterozygous at codon 280 (formerly reported as codon 119) on the *Ace*-*1* gene, coding for both the resistant (serine) and susceptible (glycine) amino acids.

**Conclusions:**

New molecular assays to amplify and screen the regions of the *VGSC* and *Ace*-*1* genes associated with insecticide resistance are reported for *An. albimanus*, *An. darlingi*, *An. vestitipennis*, and *An. pseudopunctipennis*. The development of these PCR assays presents an important advance in the analysis of target-site resistance in malaria vectors in the Americas, and will further facilitate the characterization of insecticide resistance mechanisms in these species.

## Background

Impressive gains have been made in malaria control in recent years, however, malaria remains as an endemic disease in Latin America. In 2017, a total of 976,800 confirmed cases were reported in the Americas by the World Health Organization (WHO) [1]. Currently, *Plasmodium vivax*, *Plasmodium falciparum* and *Plasmodium malariae* are the only malaria species reported in the Americas, with the vectors *Anopheles albimanus* and *Anopheles darlingi* present over a wide geographical range [1, 2]. In addition, there is ongoing malaria transmission in areas with *Anopheles vestitipennis* and *Anopheles pseudopunctipennis* as principal or secondary vectors [2, 3].

The main vector control measures used in the Americas are long-lasting insecticide-treated bed nets (LLINs) and indoor residual spraying (IRS) [1]. These measures rely on the use of insecticides, mainly pyrethroids, which are often the most cost-effective and, until recently, the only class of insecticides approved by WHO for use on LLINs [1, 4]. Given this reliance on chemical interventions, monitoring of insecticide resistance is critical. Between 2010 and 2016, a total of 55 countries with ongoing malaria transmission reported the development of pyrethroid resistance in malaria vectors [5]. In Latin America, insecticide resistance has been detected in *An*. *albimanus* populations from Peru, Ecuador, Dominican Republic, Panama, Mexico, Honduras, and Guatemala [3, 5–12]. Moreover, pyrethroid resistance has been reported in *An. darlingi* populations from Brazil, Bolivia, Peru, and Colombia [5, 13]. In the case of *An. vestitipennis* and *An. pseudopunctipennis*, resistance to DDT was reported across Latin America during 1980s [14, 15]. However, resistance to pyrethroids, carbamates and organophosphates was observed only in populations of *An. pseudopunctipennis* from Guatemala and Honduras during the same period. Despite the limited information collected between 2005 and 2019, recent surveillance data suggest that *An. vestitipennis* and *An. pseudopunctipennis* remain susceptible to all classes of insecticides in Guatemala (unpublished results, Norma Padilla), but resistance to pyrethroids was detected in a population of *An. pseudopunctipennis* from Peru [7].

Insecticide resistance in mosquitoes can be mediated by changes in insect behavior, modification in the composition of the exoskeleton or digestive tract linings, increases in enzymatic activity (metabolic resistance), or single nucleotide polymorphisms (SNPs) which produce amino acid changes on insecticide target-sites (target-site resistance) [16]. The most studied mechanisms in *Anopheles* are those related to metabolic and target-site resistance. Metabolic resistance arises from increases in the levels of major detoxification enzyme families, principally cytochrome P450s, carboxylesterases and glutathione-*S*-transferases [16–18]. In the case of target-site resistance, non-synonymous SNPs at codon 995 on the voltage-gated sodium channel (*VGSC*) gene can confer resistance to pyrethroids and DDT (referred to as ‘knockdown resistance’, or *kdr*) [19, 20]. The most common amino acid changes on the *VGSC* gene are from leucine to phenylalanine, serine, or cysteine [21]. Similarly, non-synonymous substitutions at codon 280 on the acetylcholinesterase-1 *(Ace*-*1*) gene produce an amino acid change from leucine to serine resulting in cross resistance to organophosphates and carbamates [22]. Based on the new *Anopheles gambiae* coding numbering [23, 24], codon 995 corresponds to what had previously been referred to as position 1014 (in reference to its position in the house fly *Musca domestica*) and codon 280 corresponds to what had previously been referred to as position 119 (in reference to its position in the fish *Torpedo californica*).

According to the World Health Organization’s Global Plan for Insecticide Resistance Management (GPIRM), the emergence and spread of insecticide resistance poses a major threat to malaria elimination efforts worldwide [25, 26]. The plan stresses the need to fill knowledge gaps on mechanisms of insecticide resistance in order to develop more comprehensive resistance management strategies. Managing insecticide resistance requires not only the detection of resistant phenotypes, but also the identification of the mechanisms underlying the resistance. However, the characterization of the insecticide resistance mechanisms in malaria vectors in the Americas has lagged behind those in Africa. As such, the lack of molecular tools to describe target-site resistance in malaria vectors in the Americas has impeded the development of comprehensive resistance diagnostics, which are critical for the development of resistance management plans.

A preliminary analysis of historical samples of *An. albimanus* collected across Latin America during the 1990s when insecticide resistance was frequent in field populations reported for the first time the presence of *kdr* mutations (L995F and L995C) on the *VGSC* gene [27]. Recently, non-synonymous SNPs at codon 280 (G280S) and duplication events on the *Ace*-*1* gene in field populations of *An. albimanus* from Peru were associated with cross-resistance to carbamates and organophosphates [28]. At present, molecular assays to screen the regions of the *VGSC* and *Ace*-*1* genes associated with insecticide resistance have only been described for *An. albimanus*. Herein, new molecular assays have been developed to screen for non-synonymous SNPs on regions of the *VGSC* and *Ace*-*1* genes associated with insecticide resistance in four malaria vectors from Latin America: *An. albimanus, An. darlingi, An. vestitipennis* and *An. pseudopunctipennis*.

## Methods

### Primer design

Degenerate primers were designed to amplify and sequence the regions associated with insecticide resistance on the *VGSC* and *Ace*-*1* genes. The AKDRF2 and AADKDRR2 primers were designed to amplify the *kdr* region (including codon 995) on the *VGSC* gene for *An. albimanus* (309 bp, between exons 22 and 23) and *An. darlingi* (312 bp, between exons 20 and 21). These primers were designed based on the published sequences of *An. albimanus* [GenBank: KF137581.1, APCK01001913] and *An. darlingi* [GenBank: ADMH02001922]. The AAKDRF and AAKDRR primers previously described by Lol and colleagues [27] were used to amplify the *kdr* region in *An. vestitipennis* (263 bp) and *An. pseudopunctipennis* (254 bp). For the *Ace*-*1* gene, ACE1DAF and ACE1DAR primers were designed to amplify a partial fragment of exon 4 for *An. albimanus* and exon 2 for *An. darlingi*, both fragments include the codon 280. The primers for the *Ace*-*1* gene were designed based on the sequences of *An. albimanus* [AALB002313-RA] and *An. darlingi* [ADAC000377-RA] available on VectorBase. The primer sequences are presented in Table 1.

**Table 1:** Oligonucleotide primers used to amplify the *VGSC* and *Ace*-*1* genes in Latin American malaria vectors

Gene	Species	Product size (bp)	Primers	
*VGSC*	*An. albimanus*	308	AKDRF2	AGRTGGAAYTTYACNGAYTTY
	*An. darlingi*	311	AADKDRR2	GTTCGTCTCATTATCC
	*An. vestitipennis*	263	AAKDRF	AGATGGAAYTTYACNGAYTTC
	*An. pseudopunctipennis*	254	AAKDRR	GCAANGCTAAGAANAGRTTNAG
*Ace*-*1*	*An. albimanus*	456	ACE1DAF	TAAGAAGGTGGACGTGTGGC
	*An. darlingi*		ACE1DAR	AGGGCAAGGTTCTGATCGAA

R: A/G; Y: C/T; N: A/C/G/T

### Samples and DNA extraction

For the optimization of the conventional PCR assays, wild-caught *An. albimanus* (including DNA from historical samples of this species used in previous population genetic studies [29, 30]), *An. darlingi*, *An. vestitipennis*, and *An. pseudopunctipennis* were used as DNA templates, as well as individuals from the insecticide-susceptible *An. albimanus* Sanarate laboratory strain (Table 2). Genomic DNA was extracted from mosquitoes collected during 2014–2017 and the Sanarate strain using DNAzol (Invitrogen), according to the manufacturer’s instructions with modifications. Briefly, each mosquito was grounded with 100 µL of DNAzol and resuspended in 100 µL of 1× TE buffer.

**Table 2:** Origins of the mosquito samples used for PCR validation

Gene	Species	Country	Collection date	N
*VGSC*	*An. albimanus*	Guatemala	2017	10
	*An. darlingi*	Guatemala	2014	3
		Guatemala	2017	8
		Bolivia	2015	10
	*An. pseudopunctipennis*	Guatemala	2014	10
	*An. vestitipennis*	Guatemala	2014	10
*Ace*-*1*	*An. albimanus*	Guatemala	2017	5
		Nicaragua	1998	2
		Panama	1999	1
		Venezuela	1999	1
		Ecuador	1991	1
	*An. darlingi*	Guatemala	2017	2
		Bolivia	2015	5

*N* number of mosquitoes analyzed

### PCR conditions and sequencing of the *VGSC* and *Ace*-*1* genes

The amplification of the *kdr* region of the *VGSC* gene for *An. albimanus* and *An. darlingi* was carried out in a 50 µL reaction mix containing 1× Colorless GoTaq^®^ Flexi Buffer, 1.5 mM MgCl_2_, 0.2 mM dNTPs, 2.5 µM of each primer (AKDRF2 and AADKDRR2), 1.5 U of GoTaq^®^ Hot Start Polymerase (Promega), and 2.5 to 10 ng of DNA template. The cycling conditions were 95 °C for 3 min, followed by 40 cycles of 95 °C for 45 s, 45 °C for 45 s, and 72 °C for 1 min with a final extension step at 72 °C for 5 min in a 2720 Thermal Cycler (Applied Biosystems). In the case of *An. vestitipennis* and *An. pseudopunctipennis*, the same region was amplified using the AAKDRF and AAKDRR primers with the same reaction mix and cycling conditions as mentioned above with the exception that the annealing temperature was 40 °C for *An. vestitipennis* and 45 °C for *An. pseudopunctipennis*. For the *Ace*-*1* gene, a 25 µL reaction mixture was optimized containing 1× AccuStart™ II Gel Track™ PCR SuperMix (Quantabio), 0.4 µM of each primer (ACE1DAF and ACE1DAR), and 11 to 27 ng of DNA template. The cycling conditions were 94 °C for 3 min, followed by 35 cycles of 94 °C for 30 s, 61 °C for 30 s, and 72 °C for 1 min with a final extension step at 72 °C for 10 min in a Bio-Rad T100 Thermocyler (Bio-Rad).

All PCR products were visualized on a 2% agarose gel stained with ethidium bromide and observed under UV light. Then, 50 µL of PCR product for the *VGSC* gene were purified with 10 µL of a mix containing 1× MULTI-CORE™ buffer (Promega), 1 U of Exonuclease I (Biolabs), and 1 U of TSAP (Promega). Each sample was incubated at 37 °C for 30 min and 80 °C for 20 min in a thermocycler. Purified samples for the *VGSC* gene were directly sequenced by Macrogen Inc. (Maryland, USA) with the same primers used for the PCR amplification. For the *Ace*-*1* gene, 25 µL of PCR product were purified using a Multiscreen PCR 96-well plate (Millipore), according to the manufacturer’s instructions. Sequencing of the *Ace*-*1* gene was perfomed on an ABI 3130xl Genetic Analzyer (Applied Biosystems) in the laboratory at the U.S. Centers for Disease Control and Prevention (Atlanta, USA) using the same primers as in the PCR amplification.

### Data analysis

Consensus sequences were obtained from the partial DNA sequences for the *VGSC* and *Ace*-*1* genes using the SeqMan Pro tool of DNASTAR LaserGene 11.0 suite. The identity of DNA sequences were confirmed by a BLAST against the databases of GenBank and VectorBase. The intron positions for the *VGSC* gene in *An. pseudopunctipennis* and *An. vestitipennis* were determined by comparing the sequences obtained against the reference sequences of *An. gambiae* [VectorBase: AGAP004707-RA], *An. albimanus* [VectorBase: AALB007478-RA], and *An. darlingi* [VectorBase: ADAC011160-RA]. A DNA alignment for the unique haplotypes for each species was then constructed for each gene using Clustal Omega available on the EMBL-EBI website (https://www.ebi.ac.uk/Tools/msa/clustalo/). The *An. gambiae* sequences for the *VGSC* [VectorBase: AGAP004707-RA] and *Ace*-*1* [VectorBase: AGAP001356-RA] genes were included in the DNA alignments as a reference sequence to detect SNPs. From the DNA sequences without introns, amino acid sequences were deduced and changes were detected compared to the amino acid sequences in *An. gambiae*. The consensus sequences obtained for the *VGSC* and *Ace*-*1* genes were submitted to GenBank and the accession numbers are indicated in Table 3.

**Table 3:** GenBank accession numbers of *VGSC* and *Ace*-*1* gene partial sequences of Latin American malaria vectors

Gene	Species	GenBank accession number
*VGSC*	*An*. *pseudopunctipennis*	AP: KX863726
	*An*. *vestitipennis*	AV: KX907013
*Ace*-*1*	*An. albimanus*	AA1: MK477203
		AA2: MK477204
		AA3: MK477205
	*An. darlingi*	AD2: MK477198
		AD3: MK477199
		AD4: MK477200
		AD5: MK477201
		AD6: MK477202

## Results

**Fig. 1:**
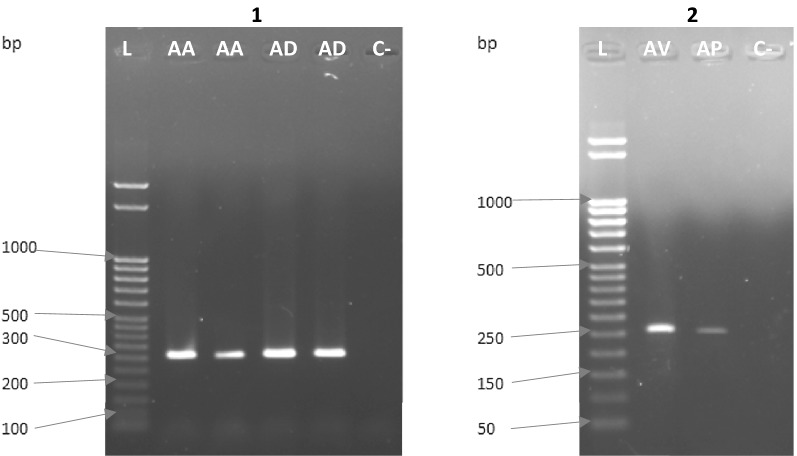
*VGSC* PCR products for malaria vectors from Latin America. (1) Agarose gel for *An*. *albimanus* (AA) and *An*. *darlingi* (AD); (2) Agarose gel for *An*. *vestitipennis* (AV) and *An*. *pseudopunctipennis* (AP). C−, negative control (water); L, ladder of 50 bp (Novagen)

**Fig. 2:**
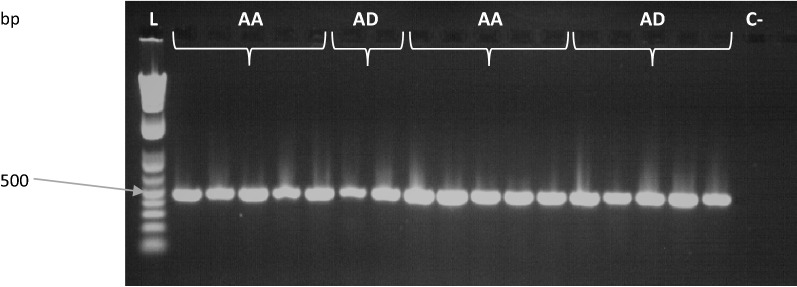
*Ace*-*1* PCR products for malaria vectors from Latin America. Agarose gel for *An*. *albimanus* (AA), and *An*. *darlingi* (AD). C−, negative control (water); L, ladder of 1 kb plus (NEB)

PCR results for the *VGSC* and *Ace*-*1* genes are presented in Figs. 1 and 2, respectively. Specific and efficient amplification was achieved for samples of *An. albimanus*, *An. darlingi, An. pseudopunctipennis*, and *An. vestitipennis* from different regions across Latin America with the described primers. In addition, DNA sequencing confirmed that the PCR products corresponded to the target areas of the *VGSC* and *Ace*-*1* genes.

**Fig. 3:**
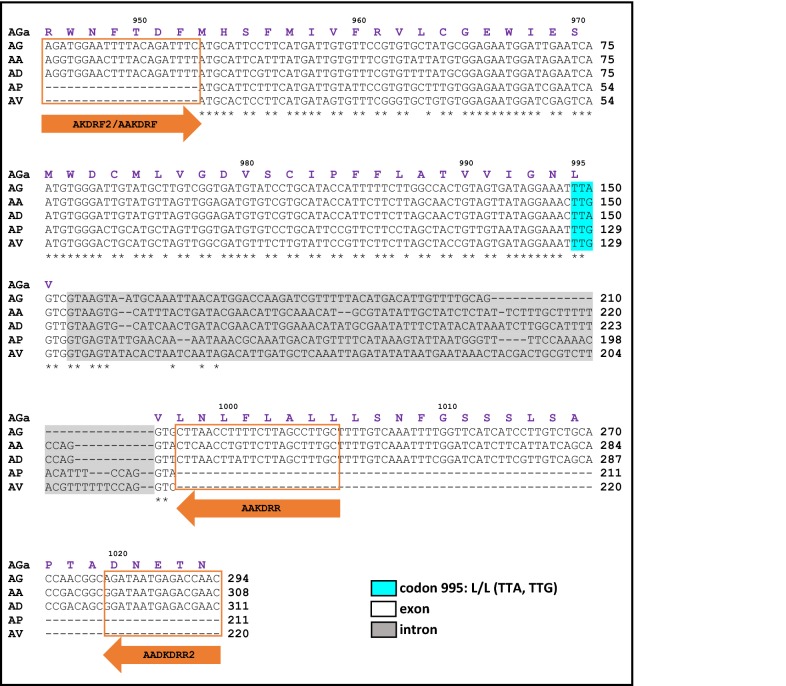
DNA alignment of the *kdr* region of *VGSC* gene of malaria vectors from Latin America. The alignment was constructed with partial DNA sequences for each species, and the aminoacid (AGa) and DNA (AG) sequences for *An. gambiae* were included as reference sequences [VectorBase: AGAP004707-RA]. Identical positions are indicated by an asterisk, primer positions are enclosed by an orange box, and the numbers above the amino acid sequence represent the codon position. Codon 995, which is associated with pyrethroid and DDT resistance (commonly reported as codon 1014, in reference to its position in the house fly *Musca domestica*), is highlighted in light blue, and it encodes the wildt-ype kdr allele (L995). AA, *An. albimanus* [VectorBase: AALB007478-RA]; AD, *An. darlingi* [VectorBase: ADAC011160-RA]; AP, *An. pseudopunctipennis* [GenBank: KX863726]; AV, *An. vestitipennis* [GenBank: KX907013]

The DNA alignment of the fragments of the *VGSC* gene showed that when different species of malaria vectors from Latin America were compared with *An. gambiae* (Fig. 3), a total of 29 SNPs were detected in *An. albimanus*, 28 in *An. darlingi*, 24 in *An. vestitipennis*, and 21 in *An. pseudopunctipennis*. Moreover, sequences obtained for *An. vestitipennis* and *An. pseudopunctipennis* were submitted to GenBank since there were no sequences available for these species. For *An. albimanus* and *An. darlingi*, sequences obtained from all samples were identical to the sequences reported in VectorBase [*An. albimanus*: AALB002313-RA; *An. darlingi* ADAC00377-RA]. The genotypes observed at codon 995 on the *VGSC* gene were TTG (*An. albimanus*, *An. pseudopunctipennis*, and *An. vestitipennis*) and TTA (*An. darlingi*), both of which code for leucine, which is associated with susceptibility to pyrethroids and DDT. All other SNPs detected on the *VGSC* gene for the analyzed species were synonymous.

**Fig. 4:**
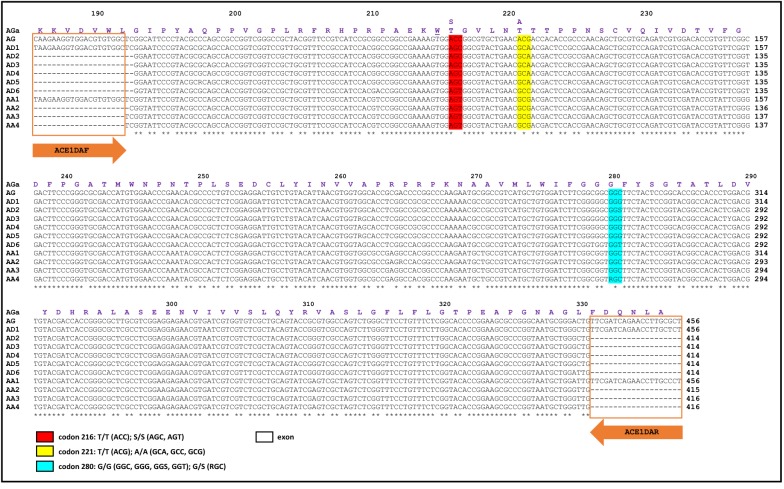
DNA alignment of the *Ace*-*1* gene for malaria vectors from Latin America. The alignment was constructed with partial DNA sequences for each species, and the aminoacid (AGa) and DNA (AG) sequences for *An. gambiae* were included as reference sequences [VectorBase: AGAP001356-RA]. Identical positions are indicated by an asterisk, primer positions are enclosed by an orange box, and the numbers above the amino acid sequence represent the codon position. Codon 280, which is associated with carbamate and organophosphate resistance (commonly reported as codon 119, in reference to its position in the fish *Torpedo californica*), is highlighted in light blue, and it encodes both homozygous susceptible (G280) and heterozygous resistant (G280S) alleles. Codons 216 and 221 are highlighted in red and yellow, respectively, and both codons result in an aminoacid change for all species from Latin American compared with the reference sequence of *An. gambiae*. Codon 215 is underlined and represents the choline binding site. Sequence AD1 corresponds to the sequence from VectorBase for *An. darlingi* [ADAC000377-RA]. Sequences AD2 [GenBank: MK477198], AD3 [GenBank: MK477199], and AD6 [GenBank: MK477202] are from Bolivia. Sequences AD4 [GenBank: MK477200] and AD5 [GenBank: MK477201] are from Guatemala. Sequence AA1 corresponds to the sequence from VectorBase for *An. albimanus* [AALB002313-RA]. Sequences AA2 [GenBank: MK477203], AA3 [GenBank: MK477204], and AA4 [GenBank: MK477205] are from Ecuador, Venezuela and Nicaragua, respectively. AA, *An. albimanus*; AD, *An. darlingi*

The DNA alignment of the fragments of the *Ace*-*1* gene from *An. darlingi* and *An. albimanus* are presented in Fig. 4. The alignment revealed 70 SNPs in *An. darlingi* and 59 SNPs in *An. albimanus,* as compared with *An. gambiae*. Based on these SNPs, five different haplotypes were detected for *An. darlingi* and three haplotypes were detected for *An. albimanus* in addition to the reference haplotypes in VectorBase for each species. At position 280 on the *Ace*-*1* gene, *An. darlingi* exhibited three potential genotypes: GGG, GGS (S = C/G), and GGT, all of which code for glycine which is associated with susceptibility to carbamate and organophosphates. However, *An. albimanus* showed the genotypes GGC (coding for glycine; associated with susceptibility) and RGC (R = A/G), which codes for both the resistant (AGC, serine) and susceptible (GGC, glycine) amino acids. Two additional non-synonymous substitutions were detected at codons 221 (threonine to alanine) and 216 (threonine to serine) on the *Ace*-*1* gene in all samples of *An. albimanus* and *An. darlingi* as compared with *An. gambiae*; these substitutions have not been previously reported before in any insect or associated with insecticide resistance [31].

## Discussion

Malaria case incidence rates decreased in Latin America by 31% between 2000 and 2015, and several countries have nearly eliminated the malaria transmission [32]. However, in recent years, many Latin American countries are increasing the use of insecticide-based vector control as part of the global push to eliminate the burden of malaria [1, 33]. This has increased the selection pressure of insecticides, favoring the emergence of insecticide resistance in malaria vectors. The continued intensive use of insecticides in commercial agriculture has also contributed to the resistance selection pressure, as was previously observed for *An. albimanus* populations from Guatemala during the 1980s [34].

Based on GPIRM recommendations, the routine monitoring of insecticide susceptibility is beginning to be included in the annual activities of National Malaria Control Programmes (NMCP) in some Latin American countries [5, 7]. However, the molecular characterization of the mechanisms underlying the resistance that has been detected is currently hindered by the lack of specific molecular assays for the principal malaria vectors of the region. Knowledge of these mechanisms is increasingly needed to better understand resistance patterns and improve vector control measures in ways that best manage and mitigate the effects of resistance. This is an important component of an Integrated Vector Management (IVM) framework, and will ultimately help elucidate the impact of insecticide resistance on malaria transmission [26]. Here, molecular assays have been developed to screen the regions of the *VGSC* and *Ace*-*1* genes associated with insecticide resistance in four malaria vectors from Latin America.

The PCR primers designed successfully amplified the target regions of both genes of interest in all samples, suggesting that results are easily replicable. Moreover, these molecular assays present the advantage of being applicable across multiple species, resulting in a potential reduction in time and costs to process samples. The DNA sequencing results showed high sequence quality in the region of interest, allowing SNPs to be identified with a high degree of accuracy. Interestingly, different susceptible genotypes were observed at key codons of the *VGSC* and *Ace*-*1* genes associated with insecticide resistance. As a consequence, these differences could limit the applicability of the allele-specific probes previously developed for African and Asian *Anopheles*. Additionally, nucleotide sequence variation in the region used to design the primes could also reduce the efficiency and specificity of the allele-specific probes developed for other *Anopheles* species. Therefore, these findings highlighting the value of this first step in characterizing important target site resistance regions in advance of developing allele-specific assays.

The molecular assays described in this paper will complement the bioassays that are required to detect resistant phenotypes. In addition to further elucidating the role of target-site mechanisms, additional research is also needed to develop molecular diagnostic tools to detect metabolic mechanisms of resistance in populations of malaria vectors from Latin America.

## Conclusions

This study reports molecular assays that amplify the regions of the *VGSC* and *Ace*-*1* genes associated with insecticide resistance in *An. albimanus*, *An. darlingi*, *An. vestitipennis* and *An. pseudopunctipennis*. These assays present an important advance in the analysis of target-site mutations in field populations of malaria vectors in Latin America, as now that these targets have been successfully amplified in these species, allele-specific diagnostic assays can be developed.

## Data Availability

All data generated or analysed during this study are included in this published article.

## Abbreviations

WHO: World Health Organization
LLINs: long-lasting insecticide-treated bed nets
IRS: indoor residual spraying
SNPs: single nucleotide polymorphisms
*VGSC*: voltage-gated sodium channel
*kdr*: knockdown resistance
*Ace*-*1*: acetylcholinesterase-1
GPIRM: Global Plan for Insecticide Resistance Management
bp: base pair
DNA: deoxyribonucleic acid
PCR: polymerase chain reaction
UV: ultraviolet
BLAST: basic local alignment search tool
NMCP: National Malaria Control Programmes
IVM: Integrated Vector Management
